# Brain-wide functional connectivity alterations and their cognitive correlates in subjective cognitive decline

**DOI:** 10.3389/fnins.2024.1438260

**Published:** 2024-08-01

**Authors:** Shaochun Huang, Siyu Wang, Zigang Che, Honglin Ge, Zheng Yan, Jia Fan, Xiang Lu, Li Liu, Wan Liu, Yeming Zhong, Caiyun Zou, Jiang Rao, Jiu Chen

**Affiliations:** ^1^Department of Rehabilitation, The Affiliated Brain Hospital of Nanjing Medical University, Nanjing, China; ^2^Fourth Clinical College, Nanjing Medical University, Nanjing, China; ^3^Department of Radiology, Nanjing Tongren Hospital, School of Medicine, Southeast University, Nanjing, China; ^4^Department of Neurosurgery, The Affiliated Brain Hospital of Nanjing Medical University, Nanjing, Jiangsu, China; ^5^Department of Human Biology, University of Cape Town Faculty of Health Sciences, Cape Town, South Africa; ^6^Department of Neurology, Northern Jiangsu People's Hospital, Clinical Medical College, Yangzhou University, Yangzhou, China; ^7^Department of Radiology, Nanjing Drum Tower Hospital, Affiliated Hospital of Medical School, Nanjing University, Nanjing, China; ^8^Institute of Medical Imaging and Artificial Intelligence, Nanjing University, Nanjing, China; ^9^Medical Imaging Center, Affiliated Drum Tower Hospital, Medical School of Nanjing University, Nanjing, China

**Keywords:** subjective cognitive decline, functional connectivity, Alzheimer's disease, brain-wide association study (BWAS), fMRI

## Abstract

**Background:**

Individuals with subjective cognitive decline (SCD) are at risk of developing Alzheimer's Disease (AD). Traditional seed-based analysis has shown biased functional connectivity (FC) in SCD individuals. To investigate unbiased altered FC by the brain-wide association study (BWAS) and to determine its association with cognition in SCD individuals.

**Methods:**

Measure of association (MA) analysis was applied to detect significant voxels with FC changes. Based on these changes, we identified regions of interest (ROIs) and conducted ROI-wise FC analyses. Correlation analyses were then performed between these FC circuits and cognition.

**Results:**

MA analysis identified 10 ROIs with significantly altered voxels. ROI-wise FC analyses revealed 14 strengthened FC, predominantly parietal-occipital link alterations. The FC between the right superior occipital gyrus and the right postcentral gyrus correlated positively with executive function, while the FC between the right middle occipital gyrus and the left angular gyrus correlated positively with episodic memory in SCD individuals.

**Conclusion:**

SCD involves multifocal impairments, of which regions of default mode network (DMN) and occipital lobe should be specially focused. Cross-hemispheric alterations indicate an internal interactive impairment pattern in SCD. The reduced FC between the right superior occipital gyrus and the right postcentral gyrus, and between the right middle occipital gyrus and the left angular gyrus, which correlate with specific cognitive functions, could serve as potential biomarkers for SCD diagnosis.

## 1 Introduction

Subjective cognitive decline (SCD) is considered a preliminary stage that is at risk of developing into Alzheimer's disease (AD) (Jessen et al., 2020). There is a growing consensus that early detection, diagnosis, and intervention of AD are crucial for potentially slowing or halting its progression (Norton et al., 2014). Therefore, gaining insights into the pathological mechanisms of SCD could deepen our understanding of AD.

Resting-state functional magnetic resonance imaging (rs-fMRI) measures intrinsic brain activities via blood-oxygen-level-dependent (BOLD) signals (Dennis and Thompson, 2014). Two meta-analyses of rs-fMRI and other neuroimaging studies in SCD have shown inconsistent functional connectivity (FC) alterations across various regions, such as the precuneus and anterior cingulate cortex (Viviano and Damoiseaux, 2020; Parker et al., 2022). These discrepancies may be due to cross-sectional designs, participant variability, and methodological differences. Seed-based analysis, commonly used to calculate FC, is indirect and site-specific, potentially introducing significant bias. Consequently, a more unbiased approach is needed to accurately detect FC patterns in SCD.

The brain-wide association study (BWAS) is inspired by the genome-wide association study (GWAS) (Savage et al., 2018). Using rs-fMRI data, BWAS investigates FC across the whole cerebrum without *a priori* region-of-interest (ROI) selection. This approach reduces the bias due to the study hypothesis and has been effectively applied in the research of depression, autism, and schizophrenia (Cheng et al., 2015, 2016; Li et al., 2017; Gong et al., 2018). Therefore, BWAS is deemed feasible for revealing early impairment in SCD.

Our study aimed to detect unbiased alterations in functional connectivity (FC) in individuals with SCD using the BWAS approach. Further correlation analyses will be conducted to reveal the association between these FC alterations and cognitive function. We hypothesize that there are unbiased alterations in FC in individuals with SCD and that these alterations are associated with cognition. Specifically, these abnormal changes in FC may serve as potential biomarkers for the early diagnosis of SCD.

## 2 Materials and methods

### 2.1 Participant recruitment

All data were obtained from the Nanjing Brain Hospital-Alzheimer's Disease Spectrum Neuroimaging Project Version 2 (NBH-ADsnp-2) (Nanjing, China) database, which is continuously updated. Detailed information regarding the NBH-ADsnp-2 database is presented in Supplementary material S.1. This study was approved by the responsible Human Participants Ethics Committee of the Affiliated Brain Hospital of Nanjing Medical University, Nanjing, China (Nos. 2018-KY010-01, 2020-KY010-02, 2021-KY029-01, 2021-KY009-01, and 2022-KY042-01). Written informed consent was obtained from all patients.

The inclusion and exclusion criteria are described in Supplementary material S.1. All participants were subjected to a standard clinical evaluation protocol. After sequencing, data on demographics and medical history were collected. Then, they underwent a comprehensive set of neurological evaluations and an MRI scan. Details regarding cognitive assessment are provided in Supplementary material S.2. Participants with excessive head-motion rotations (cumulative translation or rotation >3.0 mm or 3.0°) were excluded. For each patient, two separate neurological clinicians confirmed the diagnosis.

### 2.2 Neuropsychological assessments

The methodology employed for neuropsychological evaluations adhered to the protocols outlined in our previous research publications (Chen et al., 2015, 2016, 2019, 2020; Xu et al., 2019). The neuropsychological assessments related to cognition were conducted by three neuropsychologists. Each participant underwent a thorough and standardized neuropsychological examination that encompassed assessments of general cognitive function, executive function (EF), visuospatial function (VF), information processing speed (IPS), and episodic memory (EM). A comprehensive list of the specific neuropsychological tests administered is available in the Supplementary material S.2.

### 2.3 fMRI data acquisition and preprocessing

Detailed fMRI parameters and preprocessing procedures are shown in Supplementary material S.3, S.4, respectively. To enhance the reliability, diagnosis of each participant was conducted without the radiologists' prior knowledge. MATLAB 2015b software and the DPABI package were used to preprocess all fMRI data (Yan et al., 2016). Briefly, seven preprocessing steps were followed: (A) removal of the first 10 time points; (B) slice time correction; (C) realignment (>3 mm or >3° discarded); (D) removal of nuisance signals [i.e., white matter (WM), cerebrospinal fluid (CSF) (Chiang et al., 2012), and linear trends] by a Friston 24-parameter model; (E) spatial normalization (voxel size= 4 × 4 × 4); (F) smoothing (8 mm full-width at half-maximum); and (G) filtering (0.01–0.1 Hz). In addition to the procedures described above, we computed global intracranial volumes (ITV) using our in-house MATLAB codes based on the native gray matter (GM), WM, and CSF. This calculation was then used as a covariate in the BWAS analysis to minimize the influence of brain atrophy.

### 2.4 BWAS procession

#### 2.4.1 Voxel-w3ise FC analysis

Voxel-wise FC analysis was conducted using the automated anatomical labeling (AAL3) atlas (Rolls et al., 2020). Brain regions included in the AAL3 atlas are described in Supplementary Table S1. Each rs-fMRI image consisted of 23,178 voxels. We first extracted time series from pairwise voxels. The Pearson cross-correlation analysis was conducted at the whole-brain pairwise voxel level, followed by Fisher's z-transformation. Two-tailed two-sample *t*-tests were further applied to 268,609,842 [calculated as (23 178^*^23 178)/2] z-transformed correlational coefficients to identify significantly altered FC in individuals with SCD compared to normal controls (CN). Variables such as age, education level, framewise displacement, and ITV were regressed out as potential confounders in the analyses. To enhance statistical power and rigor, we applied a strict family-wise error correction (FWE) for multiple comparisons (Hamooya et al., 2021).

#### 2.4.2 Calculation of the measure of association

The MA value represented the strength of the association between a particular voxel and the brain disorder or condition of interest. For the voxel *i*, MA can be defined by the following formula: *MA*(*i*) = *N*_α_, where N_α_ represents the number of links between voxel *i* and every other voxel in the entire cerebrum with a *p-value* of < α. In the current study, the statistical significance was set at a *p-value* of <3.33 × 10^−2^ under FWE correction. A larger MA value indicated a more significant FC alteration in SCD. In this study, we set a threshold of MA to > 40 with a cluster size of voxels ≥ 20.

#### 2.4.3 ROI-wise FC analysis

While the MA analysis could provide information about the strength of the association between each voxel and SCD, it did not directly identify the anatomical location of the affected brain regions. To solve this problem, we further conducted an ROI-wise FC analysis. In this study, we identified brain regions with ≥20 significant voxels in the MA analysis as ROIs. We computed the average BOLD signals from the significant voxels for each ROI to extract the corresponding time series. The Pearson correlation coefficient was then applied to calculate the FC of matched ROIs. To distinguish SCD from CN, we conducted the ROI-wise FC analysis for each significant voxel. A false discovery rate (FDR) correction with a significant threshold of *p-*value of <0.05 was applied to enhance the reliability of our findings.

While the MA analysis could provide insights into the strength of association between each voxel and SCD but did not pinpoint the anatomical locations of the affected brain regions. To address this limitation, we performed an ROI-wise FC analysis, defining ROIs as brain regions containing at least 20 significant voxels from the MA analysis. We averaged the BOLD signals from these voxels for each ROI to derive the corresponding time series and calculated FC using the Pearson correlation coefficient for matched ROIs. A false discovery rate (FDR) correction with a significant threshold of *P* of <0.05 was applied to enhance the reliability of our findings.

### 2.5 Correlation analyses

Correlation analyses were conducted to measure the association between altered functional connectivity (FC) and cognition in individuals with SCD, covering four domains. To limit the impact of covariates, we used our in-house MATLAB codes to regress out the effects of age, gender, and educational level. We further applied a 10,000-time Bootstrap calculation to enhance the statistical power.

### 2.6 Robustness analysis

We executed a time-domain half-split reliability assessment to assess the robustness of the significant brain regions discovered in previous analyses utilizing the entire dataset. This involved dividing each participant's functional MRI time series into two congruent segments: the first and second halves (Cheng et al., 2015). The MA was recomputed for each segment and then analyzed separately by employing the same methodologies. Subsequently, one of these segments was designated to establish regions of interest, whereas the other segments were used for cross-validation, encompassing analyses of FC within these regions.

### 2.7 Statistical analysis

Two-sample *t*-tests were conducted on demographics, neuropsychological measures, and head rotation parameters except for gender (the chi-squared test). We further calculated z-transformed composite scores in four separate cognitive domains (i.e., EM, EF, VF, and IPS) to reduce random variability. Details of the neurological scales used in each domain along with their corresponding raw scores are available in Supplementary material S.2, Supplementary Tables S2. SPSS 22.0 was used for the analyses.

## 3 Results

### 3.1 Demographics, neuropsychological characteristics, ITV, and head motion parameters

A total of 56 individuals with SCD and 74 CNs were enrolled in this study. Differences were discovered in gender and ITV, with uncorrected *p*-values of <0.05. No significant differences were identified in terms of age, educational level, head motion parameters, scores on the Mini-Mental State Examination (MMSE), Montreal Cognitive Assessment (MoCA), or integrated Z-scores for EM, EF, IPS, or VF (all *p*-values > 0.05). However, patients with SCD scored significantly higher on the Subjective Cognitive Decline Questionnaire (SCD-Q) compared to those with CN, which aligns with the diagnostic criteria (see Table 1).

**Table 1:** Clinical characteristics among all included participants.

**Characteristics**	**CN**	**SCD**	**T-value (χ^2^)**	***p*-value**
	***n** =* **74**	***n** =* **56**		
Age (years)	63.3 (6.5)	65.6 (7.5)	−1.919	0.057
Gender (men/women)	31/43	12/44	6.030	0.014^*^
Educational level (years)	12.52 (2.64)	11.96 (2.60)	1.210	0.229
MMSE	28.58 (1.23)	28.23 (1.41)	1.033	0.304
MoCA	25.25 (2.35)	24.87 (1.90)	0.601	0.549
SCD-Q	3.76 (1.43)	6.414 (0.86)	127.308	0.000^*^
**Composite z scores for each cognitive domain**				
Episodic memory	0.22 (0.57)	0.27 (0.58)	0.108	0.914
Information processing speed	0.22 (0.71)	0.11 (0.69)	0.697	0.487
Executive function	0.22 (0.53)	0.18 (0.46)	−0.269	0.789
Visuospatial function	0.18 (0.63)	0.14 (0.64)	0.191	0.849
**Head rotation parameters**				
FD_VanDijk	0.04 (0.03)	0.05 (0.04)	−0.791	0.430
FD_Power	0.17 (0.08)	0.19 (0.13)	−1.144	0.255
FD_Jenkinson	0.08 (0.04)	0.10 (0.08)	−1.483	0.141
**Other parameters**				
ITV	1,416.22 (108.83)	1,364.83 (101.96)	2.738	0.007^*^

Data are presented as mean (standard deviation, SD). *P*-values were obtained by conducting two-sample *t* tests except for gender (the chi-squared test). We applied our in-house MATLAB code to regress out covariates (i.e., age, gender, and educational level). ^*^ Significance existed between CN and SCD subjects. Raw scores for every cognitive assessment in each domain can be found in Supplementary Table S2. CN, normal control; SCD, subjective cognitive decline; MMSE, Mini-Mental State Examination; MoCA, Montreal Cognitive Assessment; MDRS, Mattis Dementia Rating Scale; SCD-Q, Subjective Cognitive Decline-Questionnaire; HAMD, Hamilton Depression Scale; FD, framewise displacement; ITV, intracranial volumes.

### 3.2 ROIs identified by BWAS

**Figure 1:**
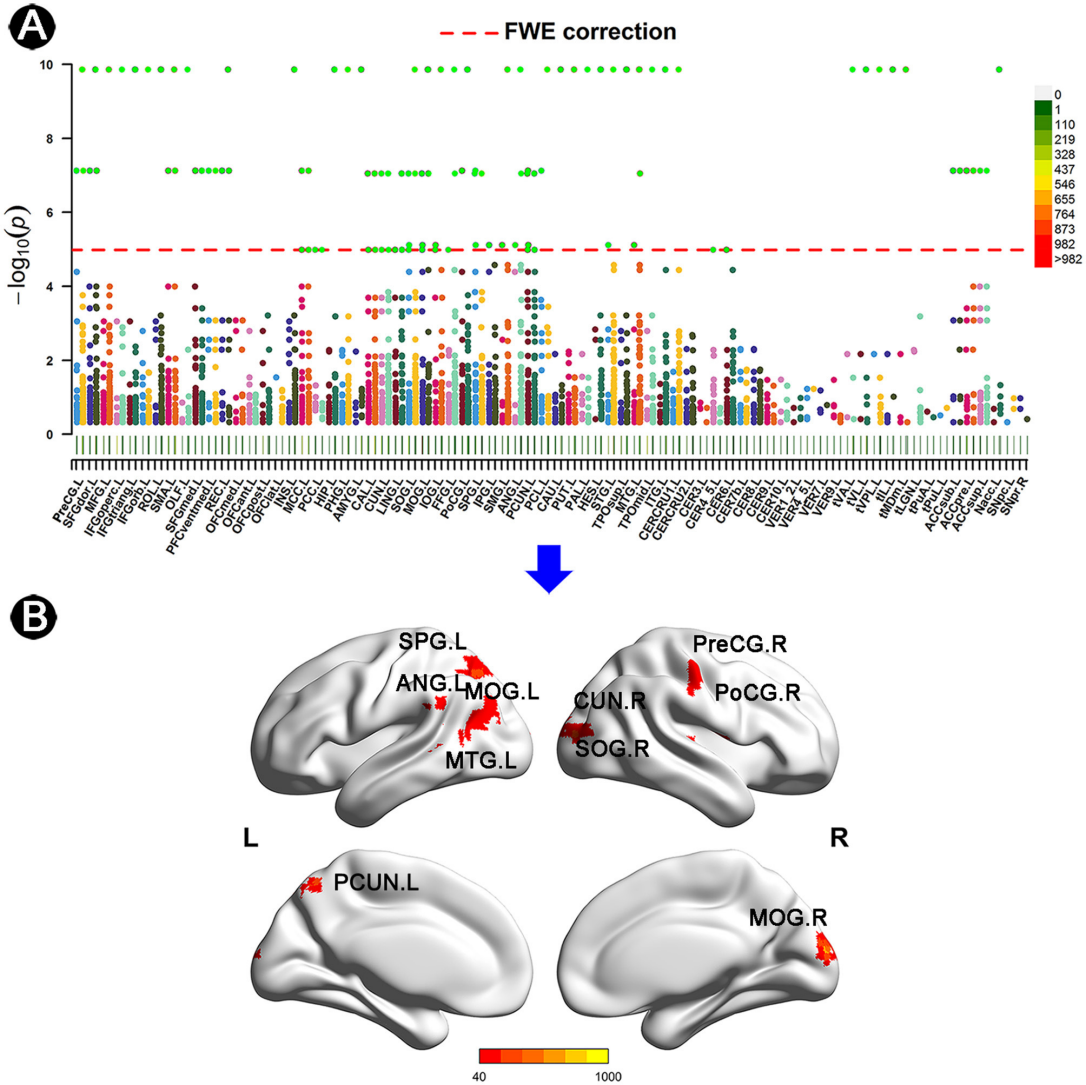
Anatomical location of altered FC in individuals with SCD. **(A)** Manhattan plot presenting the probability values of altered FC links in individuals with SCD. Every dot represents an FC link of two matched voxels. The red line indicates FWE correction with a *p-*value of <3.33 × 10^−2^. The AAL3 atlas was applied to describe the specific location of voxels, with its details listed in Supplementary material Table S1. **(B)** ROIs of ≥20 voxels with the threshold of MA of >40 after FWE correction. The color bar represents the values of MA. FC, functional connectivity; SCD, subjective cognitive decline; CN, normal control; AAL, automated anatomical labeling; FWE, family-wise error; SPG, superior parietal gyrus; ANG, angular gyrus; MOG, middle occipital gyrus; MTG, middle temporal gyrus; CUN, cuneus; SOG, superior occipital gyrus; PreCG, precentral gyrus; PoCG, postcentral gyrus; PCUN, precuneus; L, left; R, right.

Based on the AAL3 atlas, voxels with significantly altered FC were detected after FWE correction (Figure 1A). Furthermore, 10 ROIs with ≥20 significant voxels were identified (Figure 1B), with their location, number of voxels, coordinates, and peak MA values presented in Table 2. To summarize, two ROIs were located in the frontal or temporal lobes [i.e., the right precentral gyrus (PreCG.R) and the left middle temporal gyrus (MTG.L)]. Four ROIs were located in the occipital lobe [i.e., the right cuneus (CUN.R), the bilateral middle occipital gyrus (MOG.L/R), and the right superior occipital gyrus (SOG.R)], while the remaining ROIs were located in the parietal lobe [i.e., the right postcentral gyrus (PoCG.R), the left superior parietal gyrus (SPG.L), the left precuneus (PCUN.L), and the left angular gyrus (ANG.L)].

**Table 2:** Significant ROIs in BWAS.

**No**.	**L/R**	**Regions**	**Voxels in ROI**	**Peak MA value**	**MNI (Peak)**		
**CN vs. SCD**							
ROI1	R	Precentral_R	24	752	46	−18	68
ROI2	R	Cuneus_R	25	541	6	−94	16
ROI3	R	Occipital_Sup_R	21	360	14	−98	16
ROI4	L	Occipital_Mid_L	40	190	−34	−66	28
ROI5	R	Occipital_Mid_R	28	223	30	−94	16
ROI6	R	Postcentral_R	20	116	50	−22	48
ROI7	L	Parietal_Sup_L	66	282	−14	−62	44
ROI8	L	Angular_L	20	105	−38	−54	24
ROI9	L	Precuneus_L	23	304	−10	−62	52
ROI10	L	Temporal_Mid_L	55	249	−46	−58	4

Positive MA values indicated strengthened FC in CN compared to individuals with SCD. ROI, region of interest; BWAS, brain-wide association study; MA, measure of association; MNI, Montreal Neurological Institute; CN, normal control; SCD, subjective cognitive decline; FC, functional connectivity; AAL, automated anatomical labeling.

### 3.3 Altered FC patterns

**Figure 2:**
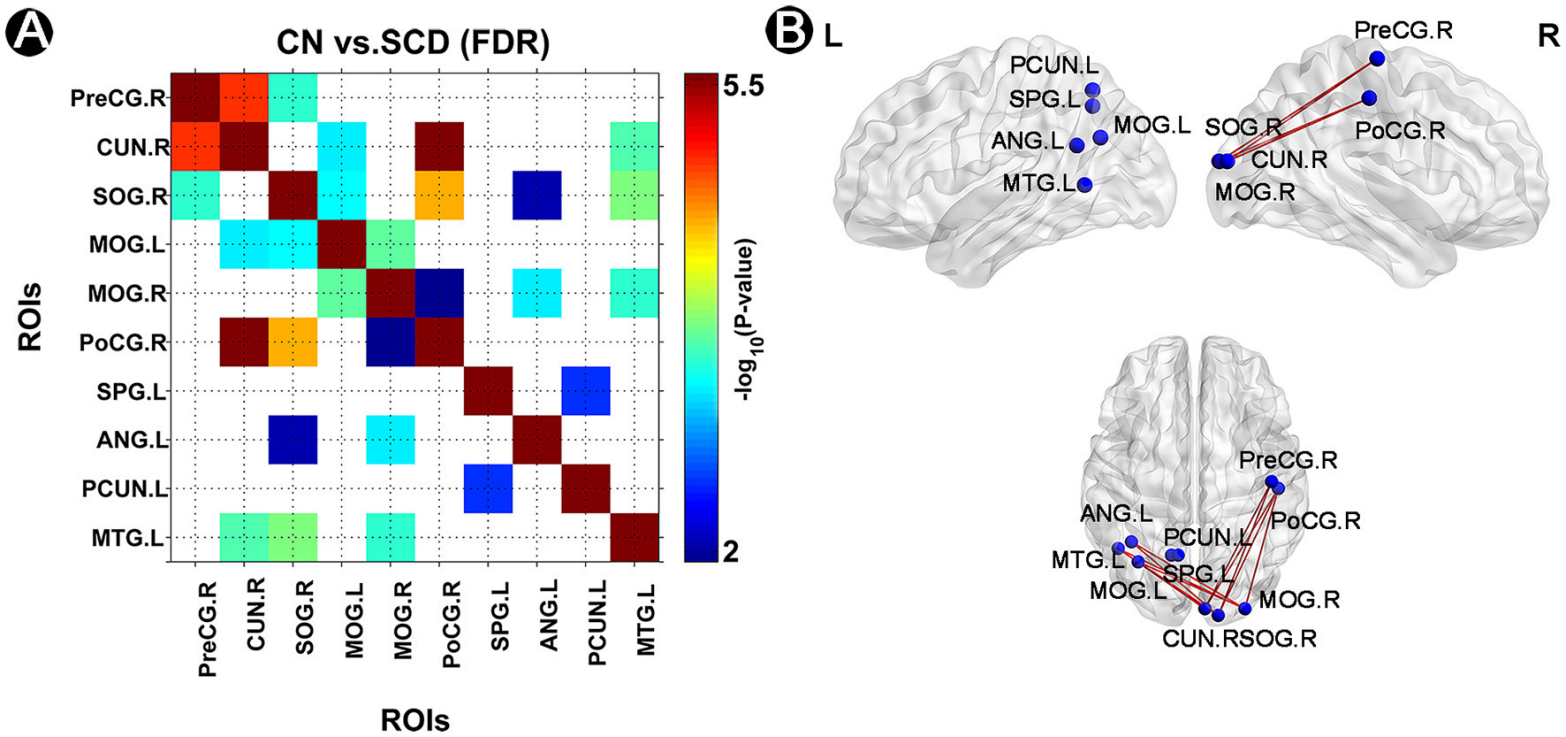
Altered FC pattern in individuals with SCD and CN. **(A)** A matrix of strengthened FC links in CN compared to individuals with SCD after FDR correction (*p* < 0.05). The *p-*value was presented in the color bar. **(B)** A brief diagram of significant FC in CN compared to individuals with SCD. FC, functional connectivity; CN, normal control; SCD, subjective cognitive decline; SPG, superior parietal gyrus; ANG, angular gyrus; MOG, middle occipital gyrus; MTG, middle temporal gyrus; CUN, cuneus; SOG, superior occipital gyrus; PreCG, precentral gyrus; PoCG, postcentral gyrus; PCUN, precuneus; L, left; R, right.

In this study, we detected 14 significant FC in CN compared to individuals with SCD after FDR correction (*p* < 0.05) (Figure 2). In brief, the left precuneus was significantly linked to the left SPG (**Circuit 1**). The left ANG was significantly associated with the right SOG and the MOG (**Circuits 2–3**). The left MTG was significantly linked to the right SOG, the right cuneus, and the right MOG (**Circuits 4–6**). The right SOG and the right cuneus were both significantly linked to the right PreCG, the left MOG, and the right PoCG (**Circuits 7–12**). The right MOG was significantly associated with the left MOG and the right PoCG (**Circuits 13–14**).

### 3.4 The robustness of the results

Supplementary Figures 2, 3 show the voxel locations in the brain where notable differences in FC between SCD and CN are observed across both datasets (FWE correction, 0.05, MA 40, cluster size 20 voxels). These figures emphasize the regions showing variability in FC in the first and second halves of the dataset. The analysis revealed that the most significant regions displayed consistency across both halves.

### 3.5 Correlation between significant FC and cognition

**Figure 3:**
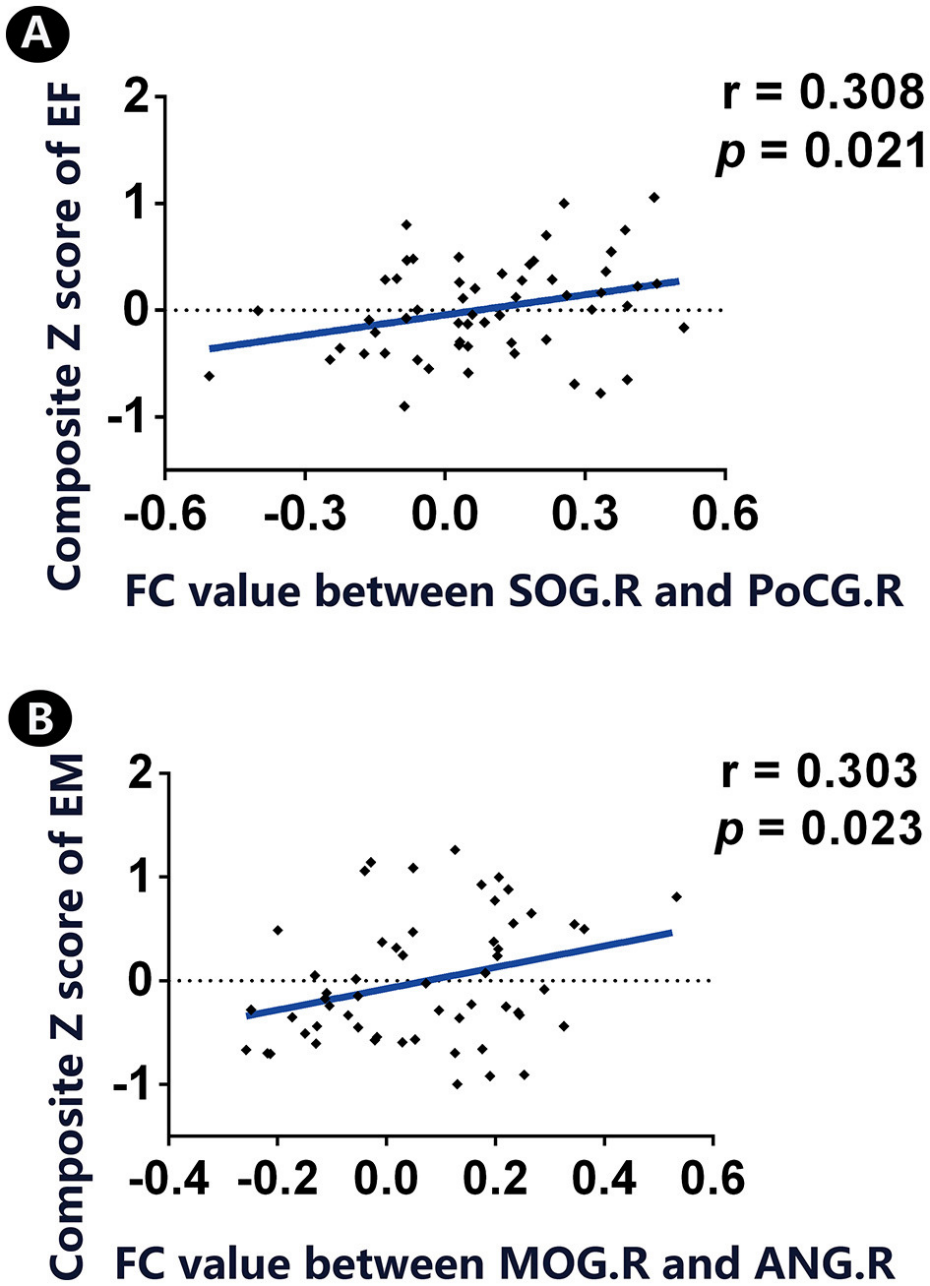
Correlation between significant FC links and cognition in individuals with SCD. **(A)** A positive association was found between significant FC between SOG.R and PoCG.R and the z-transformed composite EF score. **(B)** There is a positive association between significant FC between MOG.R and ANG.L and the z-transformed composite EM score. FC, functional connectivity; CN, normal control; SCD, subjective cognitive decline; SOG, superior occipital gyrus; PoCG, postcentral gyrus; MOG, middle occipital gyrus; ANG, angular gyrus; EM, episodic memory; EF, executive function; R, right; L, left. Throughout the analyses, we applied our in-house MATLAB code to regress out covariates (i.e., age, gender, and educational level).

The FC circuit between the right SOG and right PoCG shared a positive association with EF. Meanwhile, the FC circuit of the right MOG and left ANG was positively correlated with EM (Figure 3).

## 4 Discussion

To the best of our knowledge, this is the first study to reveal unbiased FC alterations in SCD through BWAS. We conducted correlation analyses to explore significant associations between altered FC and cognition. Our findings revealed multi-site impairments, predominantly in the parietal and occipital lobes, with two weakened FCs correlating with poorer performance in EM and EF. Overall, this study provides insights into the pathological mechanisms of SCD and identifies the potential biomarkers for SCD detection.

### 4.1 Significant FC in regions of the default mode network

The precuneus, located in the medial parietal lobe, is a core region of the default mode network (DMN) involved in episodic memory retrieval and spatial cognition (Cavanna and Trimble, 2006). Additionally, the ANG, part of the DMN, is situated in the temporal lobe and contributes to episodic and semantic cognition (Humphreys et al., 2021). The meta-analysis conducted by Viviano and Damoiseaux (2020) concluded that FC alterations in SCD primarily occur in DMN regions and medial temporal structures. We detected significant FC between the left precuneus and the left SPG, as well as between the left ANG and the right SOG/MOG. This finding aligns with previous findings that highlight the DMN's critical role in SCD progression.

Furthermore, we observed a weakened FC between the MOG and ANG, which correlated with the EM decline in individuals with SCD. The hallmark of SCD is the self-reported memory decline, although cognitive performance does not significantly deteriorate. However, FC alterations begin early and may predict potential cognitive dysfunction as the disease progresses.

### 4.2 Significant FC in the MTG

The MTG is located in the temporal lobe and plays a significant role in social cognition, comprehension, and semantic processing (Xu et al., 2019; Lukic et al., 2021). Impairment in the MTG is commonly found in AD patients (Govindpani et al., 2020). Our results indicate that alterations in the MTG are detectable even in individuals with SCD. We have observed altered FC between the MTG and the occipital lobe (i.e., SOG and MOG) and the parietal lobe (i.e., cuneus). This FC reflects the complex mechanisms of cross-regional damage. These results suggest that the MTG could serve as a potential biomarker for diagnosing AD and its preclinical stages.

### 4.3 Trend of multi-site FC impairment across the whole cerebrum

In addition to the FC impairment mentioned above, the right cuneus was significantly linked to the right PreCG, left MOG, and right PoCG. The cuneus, which is adjacent to the precuneus, is located on the medial surface of the occipital lobe. As for previous studies, Matías-Guiu et al. (2017) have reported brain atrophy in this region. Furthermore, Kawagoe et al. (2019) have found increased connectivity between the cuneus and the occipital lobe. De Marco et al. (2017) mentioned that studies reporting deregulation in the cuneus also provided insights into the occipital physiology.

However, our findings indicate a reduction in the FC circuit for individuals with SCD. This discrepancy may be attributed to variations in the time since the onset of SCD (Viviano and Damoiseaux, 2020). Participants with a shorter onset duration often exhibit strengthened FC due to potential compensatory mechanisms, whereas those with prolonged SCD often exhibit decreased FC, which is indicative of neurodegeneration and greater physical dysconnectivity. Our results highlight the role of the cuneus in the pathological progression of SCD.

Both the MOG and the SOG are part of the occipital lobe. In our study, we identified altered FC in the SOG with the MTG, PreCG, PoCG, and ANG. Regarding the MOG, altered FC was detected with the MTG, ANG, and cuneus. The occipital lobe is known as the cortical region, where alpha rhythms are prominent at rest, particularly with the eyes closed (Cohn, 1948). These alpha rhythms in the occipital lobe further positively correlate with GM density and cognitive function in the preclinical stages of AD (Babiloni et al., 2015). Specifically, the disruption of the DMN often occurs within these alpha rhythms in amnestic mild cognitive impairment, which is considered a more advanced stage of SCD (Garcés et al., 2014). Similar to previous studies, our study confirms the significance of the occipital lobe in SCD.

In addition to the MTG, ANG, and cuneus, as described above, PreCG and PoCG held significant FC links with the MOG or the SOG. The PreCG, located in the posterior part of the frontal lobe, is involved in verbal short-term memory and linguistic functions.

Previous studies have interpreted altered fronto-occipital FC as a compensatory mechanism in AD and for task switching in older adults (Hakun et al., 2015; De Marco et al., 2017). However, our findings indicated impairments in these regions. We hypothesize that this may be due to the long-term process of SCD, which can lead to the breakdown of compensatory mechanisms.

Alternatively, it might represent a compensatory response of the occipital lobe to downgrade FC and counteract impairment. The PoCG is commonly regarded as the region possessing sensory functions (Nelson and Chen, 2008). In pre-AD stages, reduced spontaneous brain activities have been detected in the PoCG (Zeng et al., 2019). Moreover, EFs were positively linked to the FC circuit of the SOG and PoCG, indicating that these two regions are involved in advanced cognitive functions. Overall, the occipital lobe and its connections with other regions may serve as a core region in SCD.

In conclusion, there may exist a trend of multi-site FC impairment across the whole cerebrum. In addition to focusing on the pre-cuneus, ANG, and MTG, it is crucial to pay significant attention to the occipital lobe and the cuneus in individuals with SCD. Upon reviewing the results comprehensively, we were particularly struck by the significant FC observed across both hemispheres. Specifically, the right MOG displayed altered FCs with the left ANG and the left MTG, while the left MOG showed altered FCs with the right cuneus and the right SOG. This cross-hemispheric phenomenon suggests the existence of a potential interactive impairment mechanism within the bilateral cerebrum.

In addition, our study has successfully identified specific brain regions that exhibit abnormalities in individuals with SCD. These regions, which include the pre-cuneus, the middle temporal gyrus, and several other key areas, are critical to the pathophysiology of AD.

Identifying these abnormal brain regions in individuals with SCD provides a promising avenue for targeted interventions. Early intervention strategies could potentially mitigate the progression of AD by addressing these specific areas of neural dysfunction. Future research should focus on developing and testing interventions targeting these specific brain regions in individuals with SCD. The interventions could include pharmacological treatments, cognitive training programs, or other therapeutic approaches tailored to the early stages of AD.

This study represents the first application of the unbiased BWAS method in individuals with SCD, providing insights into the early impairment patterns of SCD. However, this study has several limitations. First, the study employs a cross-sectional design, which may not fully capture the underlying pathological mechanisms of disease progression. Future studies will benefit from a longitudinal perspective, which will be facilitated as our NBH-ADsnp-2 database continues to be updated with participant follow-ups. Second, significant differences in gender and ITV were observed between CN and SCD subjects; these factors were included as covariates to mitigate their impact. Third, the diverse definitions of SCD can lead to inconsistencies across studies; our study utilized the SCD-Q assessment to distinguish between SCD patients and CN.

Additionally, while the AAL atlas provides a robust anatomical framework, future studies may benefit from incorporating a functional zoning map designed to delineate regions based on their functional properties and connectivity patterns, offering a more nuanced understanding of brain function. Finally, the correlation between altered FC and cognition was not corrected for multiple comparisons, necessitating caution in drawing definitive conclusions.

## 5 Conclusion

We can conclude that SCD exhibits a pathophysiological pattern characterized by multi-site impairment, with the DMN being particularly implicated. Special attention should be directed toward the occipital lobe, as the altered FC observed across both hemispheres suggests an underlying interactive deteriorative pattern in SCD individuals. Specific FC patterns associated with abnormal cognitive domains may serve as potential biomarkers for the early detection and diagnosis of SCD.

## Data availability statement

The raw data supporting the conclusions of this article will be made available by the authors, without undue reservation.

## Ethics statement

The studies involving humans were approved by the responsible Human Participants Ethics Committee of the Affiliated Brain Hospital of Nanjing Medical University, Nanjing, China (Nos. 2018-KY010-01, 2020-KY010-02, 2021-KY029-01, 2021-KY009-01, and 2022-KY042-01). The studies were conducted in accordance with the local legislation and institutional requirements. The participants provided their written informed consent to participate in this study.

## Author contributions

SH: Data curation, Writing – original draft. SW: Data curation, Formal analysis, Writing – original draft. ZC: Data curation, Writing – original draft. HG: Data curation, Writing – original draft. ZY: Data curation, Writing – original draft. JF: Formal analysis, Writing – original draft. XL: Data curation, Writing – original draft. LL: Data curation, Writing – original draft. WL: Data curation, Writing – original draft. YZ: Data curation, Writing – original draft. CZ: Data curation, Writing – original draft. JR: Conceptualization, Writing – review & editing. JC: Conceptualization, Formal analysis, Funding acquisition, Validation, Writing – review & editing.
