# Supplementary material for: Brain-wide functional connectivity alterations and their cognitive correlates in subjective cognitive decline
Source: Front Neurosci. 2024 Aug 1;18:1438260. doi: 10.3389/fnins.2024.1438260 (PMC11324595; doi:10.3389/fnins.2024.1438260)
Supplement: Supplementary file 1 [file Data_Sheet_1.docx]

# Brain-wide functional connectivity alterations associated with cognition in subjective cognitive decline.

**Supporting Information**

**Material and method**

**S.1** **NBH-ADsnp-2 database**

Data used in this study were obtained from the Nanjing Brain Hospital-Alzheimer’s Disease Spectrum Neuroimaging Project Version 2 (NBH-ADsnp-2) database. NBH-ADsnp-2 is an upgrade of NBH-ADsnp and is derived from an Alzheimer’s Disease Spectrum Neuroimaging cooperative Project that was jointly built in September 2022 by Department of Radiology, Nanjing Drum Tower Hospital and Department of Radiology and Neurology, the Affiliated Brain Hospital of Nanjing Medical University. Prof. Jiu Chen, PhD, MD, from Nanjing Drum Tower Hospital, and Xingjian Lin, MD and Chaoyong Xiao, MD, from the Affiliated Brain Hospital of Nanjing Medical University, acts as the principal investigator of NBH-ADsnp-2. NBH-ADsnp-2 was initiated by Dr. Jiu Chen, Dr. Xingjian Lin, and Dr. Chaoyong Xiao and was named by Dr. Chen, Dr. Lin, Dr. Xiao's cooperative research group (discussed by Chen Xue, Guan-jie Hu, Wen-wen Xu, Wan Liu, Wen-zhang Qi, Si-yu Wang, Jia-ni Xu, Shan-shan Chen, Honglin Ge, Zheng Yan, Yu Song, Qianqian Yuan, Huimin Wu, Xuhong Liang, Xinyi Yang and finally verified by Jiu Chen, Xingjian Lin, and Chaoyong Xiao). NBH-ADsnp-2 is an observational and intervention study which includes cross-sectional and longitudinal follow-up components. The goal of NBH-ADsnp-2 is to identify early neuroimaging biomarkers of preclinical Alzheimer’s Disease (AD) spectrum (subjective cognitive decline (SCD), amnestic mild cognitive impairment (aMCI), amnestic mild cognitive impairment (naMCI), and AD), to predict the disease progression of individuals within preclinical AD spectrum, and to provide imaging-based targets for individualized intervention in order to prevent the disease deterioration from preclinical stages to the eventually progressed AD. All subjects in NBH-ADsnp-2, who were all Han Chinese and right-handed, were recruited initially from hospitals and local communities by advertising and by means of broadcasting. This database used a standardized clinical evaluation protocol that included a medical history interview, neurologic examination, a battery of neurocognitive assessment, and resting-state MRI scan (T1, T2, 3D T1, DTI, and BOLD) for all participants (healthy controls, SCD, naMCI, aMCI, and AD). In addition, MRI data collected after 2 and 4 weeks of rTMS intervention in patients with SCD and MCI were added after the database upgrade. All subjects and their study partners completed the informed consent process, and the study protocols were reviewed and approved by the responsible Human Participants Ethics Committee of the Affiliated Brain Hospital of Nanjing Medical University (No. 2018-KY010-01, No. 2020-KY010-02, No.2021-KY029-01, No. 2021-KY009-01, No. 2022-KY042-01, No. ChiCTR2000034533, No. ChiCTR1900022287).

The general eligibility, inclusion, and exclusion criteria for NBH-ADsnp-2 subjects can be found as follows:

Inclusion criteria of SCD subjects were identified meeting the published SCD research criteria proposed by the Subjective Cognitive Decline Initiative (SCD-I) (Jessen et al., 2014), and the detailed inclusion criteria have been described in our previously published studies (Xue et al., 2019), as follows: (a) self-reported persistent memory decline, which was confirmed by an informant; (b) Subjective Cognitive Decline Questionnaire (SCD-Q) score > 5 (Hao et al., 2017;Yan et al., 2018;Cedres et al., 2019); (c) performance within the normal range on MMSE and MoCA (adjusted for age and education); (d) Clinical Dementia Rating (CDR) = 0; and (e) subjects aged between 50 and 80 years old.

Inclusion criteria of naMCI subjects were referenced to previous studies (Dunn et al., 2014;Xue et al., 2019), as follows: a) normal overall cognitive function just like aMCI; b) the tests scores about memory function are in the normal range while deficits were present on other cognitive domains including visual spatial function, executive function, language function, and information processing speed; and (c) subjects aged between 50 and 80 years old.

Inclusion criteria of aMCI subjects were identified meeting the diagnostic criteria defined by Peterson et al. (Petersen et al., 1999) as well as the revised consensus standards presented by Winblad et al. (Winblad et al., 2004), and the detailed inclusion criteria have been described in our previously published studies (Chen et al., 2016b;Chen et al., 2019a;Xue et al., 2019;Chen et al., 2020;Chen et al., 2022), as follows: (a) memory complaint preferably corroborated by an informant or the subject for more than 3 months; (b) objective memory impairment adjusted for age and educational level; (c) normal general cognitive function of MMSE score equal or above 24; (d) no or minimal impairment in daily living activities; (e) CDR=0.5; (f) subjects aged between 50 and 80 years old; and (g) absence of dementia symptoms that were not sufficient to meet the criteria of the National Institute of Neurological and Communicative Disorders and Stroke or the AD and Related Disorders Association criteria for AD.

Inclusion criteria of CN subjects were identified meeting the following rules: (a) without memory complaint; (b) normal cognitive performance matched with age and education; (c) CDR=0; (d) MMSE ≥ 26; and (e) subjects aged between 50 and 80 years old. (Chen et al., 2019b;Xue et al., 2019;Chen et al., 2020;Chen et al., 2022).

The detailed exclusion criteria for all subjects have been described in our previously published studies (Chen et al., 2016b;Chen et al., 2019a;Xue et al., 2019;Chen et al., 2020;Chen et al., 2022), as follows: (a) a past history of stroke (modified Hachinski Ischemic Scale Score of > 4), alcoholism, head injury, brain tumors, Parkinson’s disease, epilepsy, encephalitis, major depression (excluded by HAMD), or other neurological or psychiatric illness (excluded by clinical assessment and case history); (b) major medical illness (e.g., cancer, anemia, thyroid dysfunction, syphilis, or HIV); (c) severe visual or hearing loss; (d) unable to complete neuropsychological tests or with a contraindication for MRI, and (5) T2-weighted MRI showing major white matter (WM) changes, infarction, or other lesions (two experienced radiologists analyzed the scans). All patients didn’t have any medications.

**S.2 Neuropsychological assessments for the NBH-ADsnp-2 database**

Neuropsychological assessments were as described in our previously published studies (Chen et al., 2015;Chen et al., 2016a;Chen et al., 2019a;Chen et al., 2019b;Xue et al., 2019). All subjects underwent a standardized clinical interview and comprehensive neuropsychological assessments that were performed by neuropsychologists (Dr. Xue, Qi, and Liu), including Mini Mental State Examination (MMSE), Montreal Cognitive Assessment (MoCA), Mattis Dementia Rating Scale (MDRS), Auditory Verbal Learning Test - immediate recall (AVLT-IR), Auditory Verbal Learning Test-5-min delayed recall (AVLT-5-min-DR), Auditory Verbal Learning Test-20-min delayed recall (AVLT-20-min-DR), Logical Memory Test-immediate recall (LMT-IR), Logical Memory Test-20-min delayed recall (LMT-20-min-DR), Rey-Osterrieth Complex Figure Test-20-min delayed recall (ROCFT-20min-DR), Clock Drawing Test (CDT), Rey-Osterrieth Complex Figure Test (ROCFT), Verbal Fluency Test (VFT), Digit Span Test (DST), Digital Symbol Substitution Test (DSST), Trail-Making Tests A and B (TMT-A and B), Stroop Color and Word Test A, B, and C, and Semantic Similarity (Similarity) test. These tests were used to evaluate general cognitive function, episodic memory, information processing speed, executive function, and visuo-spatial function, respectively.

**S.3 Image acquisition for the NBH-ADsnp-2 database**

The NBH-ADsnp data acquisition process was as described in our previously published studies (Chen et al., 2016b;Chen et al., 2019a;Chen et al., 2019c). The details regarding image acquisition parameters in NBH-ADsnp were provided in our previously published study(Xue et al., 2019).

All MRI data were acquired using a 3.0 Tesla Verio Siemens scanner with an 8-channel head-coil in the Affiliated Brain Hospital of Nanjing Medical University (Nanjing, China). Resting-state functional images were collected when participants were instructed to rest with their eyes open, to not fall asleep, and to not think of anything in particular. The gradient-echo echo-planar imaging (GRE-EPI) sequence included 240 volumes. The parameters were as follows: repetition time (TR) = 2000 ms, echo time (TE) = 30 ms，number of slices = 36, thickness = 4.0 mm, gap = 0 mm, matrix = 64×64, flip angle (FA) = 90°, field of view (FOV) = 220 mm×220 mm, acquisition bandwidth = 100 kHz, voxel size = 3.4×3.4×4 mm^3^. The imaging process took approximately 8 minutes.

High-resolution T1-weighted images were acquired by a 3D magnetization-prepared rapid gradient-echo (MPRAGE) sequence. The parameters were as follows: TR = 1900 ms, TE = 2.48 ms, inversion time (TI) = 900 ms, number of slices = 176, thickness = 1.0 mm, gap = 0.5 mm, matrix = 256×256, FA = 9°, FOV = 256 mm × 256 mm, voxel size = 1×1×1 mm^3^. The imaging process took approximately 4.26 minutes.

Additionally, routine axial T2-weighted images were acquired to rule out subjects with major changes in WM, cerebral infarction or other lesions using flair sequence as below: TR = 8400 ms, TE = 94 ms, FA= 150°, acquisition matrix = 256×256, FOV = 230×230 mm, thickness = 5.0 mm, gap = 0 mm, and number of slices = 20. The imaging process took approximately 2.50 minutes.

**S.4 fMRI image preprocessing**

All fMRI data were preprocessed using MATLAB2015b (http://www.mathworks.com/products/matlab/) and DPABI image processing software (Yan et al., 2016). The image processing procedure was as previously described (Yan et al., 2013) and as follows: the first ten volumes were discarded to reduce the instability of MRI signals. Corrections were performed for the intra-volume acquisition time differences among slices and inter-volume motion effects during the scan (slice timing correction and head motion correction) (Power et al., 2012;Van Dijk et al., 2012). Participants with excessive head motion (cumulative translation or rotation > 3.0 mm or 3.0°) were excluded. Individual functional and structural images were co-registered. The Diffeomorphic Anatomical Registration Through Exponentiated Lie Algebra (DARTEL) algorithm was used to normalize and segment the structural images into gray matter (GM), WM and cerebrospinal fluid (CSF) partitions (Ashburner and Friston, 2009). We next used a Friston 24-parameter model (*i.e.*, 6 head motion parameters, 6 head motion parameters one time point before, and the 12 corresponding squared items) to regress out head motion effects from the realigned data (Friston et al., 1996). The WM, CSF, and the linear trends were also regressed as nuisance covariates (Brady et al., 2019). After realigning, slice timing correction, and co-registration, framewise displacement (FD) was calculated for all resting state volumes (Power et al., 2012). All volumes with a FD_Jenkinson greater than 0.2 mm were regressed out as nuisance covariates (Brady et al., 2019). Any scan with 50% volumes removed was discarded (Brady et al., 2019). After nuisance covariate regression, functional images were normalized by DARTEL into MNI space (resampling voxel size, 4 × 4 × 4 mm³) and then spatially smoothed by a Gaussian kernel of 8 mm full-width at half maximum (FWHM) to reduce spatial noise. Temporal band-pass filtering (0.01–0.1 Hz) was applied to reduce the effect of low-frequency drifts and high-frequency physiological noise. Voxels within a group GM mask created by DARTEL were used for further analyses.

**SI Tables**

**Table S1. The name and abbreviation of the regions of interest (ROIs) based on the AAL3v1 atlas (Rolls et al., 2020)**

| **NO.** | **Regions** | **Abbr.** | **NO.** | **Regions** | **Abbr.** |
| --- | --- | --- | --- | --- | --- |
| 1, 2 | Precentral gyrus | PreCG | 91,92 | Temporal pole: middle | TPOmid |
| 3, 4 | Superior frontal gyrus, dorsolateral | SFGdor | 93,94 | Inferior temporal gyrus | ITG |
| 5, 6 | Middle frontal gyrus | MFG | 95,96 | Crus I of cerebellar hemisphere | CERCRU1 |
| 7, 8 | Inferior frontal gyrus, opercular part | IFGoperc | 97,98 | Crus II of cerebellar hemisphere | CERCRU2 |
| 9, 10 | Inferior frontal gyrus, triangular part | IFGtriang | 99,100 | Lobule III of cerebellar hemisphere | CER3 |
| 11, 12 | IFG pars orbitalis | IFGorb | 101,102 | Lobule IV, V of cerebellar hemisphere | CER4_5 |
| 13, 14 | Rolandic operculum | ROL | 103,104 | Lobule VI of cerebellar hemisphere | CER6 |
| 15, 16 | Supplementary motor area | SMA | 105,106 | Lobule VIIB of cerebellar hemisphere | CER7b |
| 17, 18 | Olfactory cortex | OLF | 107,108 | Lobule VIII of cerebellar hemisphere | CER8 |
| 19, 20 | Superior frontal gyrus, medial | SFGmed | 109,110 | Lobule IX of cerebellar hemisphere | CER9 |
| 21, 22 | Superior frontal gyrus, medial orbital | PFCventmed | 111,112 | Lobule X of cerebellar hemisphere | CER10 |
| 23, 24 | Gyrus rectus | REC | 113 | Lobule I, II of vermis | VER1_2 |
| 25, 26 | Medial orbital gyrus | OFCmed | 114 | Lobule III of vermis | VER3 |
| 27, 28 | Anterior orbital gyrus | OFCant | 115 | Lobule IV, V of vermis | VER4_5 |
| 29, 30 | Posterior orbital gyrus | OFCpost | 116 | Lobule VI of vermis | VER6 |
| 31, 32 | Lateral orbital gyrus | OFClat | 117 | Lobule VII of vermis | VER7 |
| 33, 34 | Insula | INS | 118 | Lobule VIII of vermis | VER8 |
| 35, 36 | Anterior cingulate & paracingulate gyri | ACG | 119 | Lobule IX of vermis | VER9 |
| 37, 38 | Middle Cingulate & paracingulate gyri | MCC | 120 | Lobule X of vermis | VER10 |
| 39, 40 | Posterior cingulate gyrus | PCC | 121,122 | Thalamus, Anteroventral Nucleus | tAV |
| 41, 42 | Hippocampus | HIP | 123,124 | Lateral posterior | tLP |
| 43, 44 | Parahippocampal gyrus | PHG | 125,126 | Ventral anterior | tVA |
| 45, 46 | Amygdala | AMYG | 127,128 | Ventral lateral | tVL |
| 47, 48 | Calcarine fissure & surrounding cortex | CAL | 129,130 | Ventral posterolateral | tVPL |
| 49, 50 | Cuneus | CUN | 131,132 | Intralaminar | tIL |
| 51, 52 | Lingual gyrus | LING | 133,134 | Reuniens | tRe |
| 53, 54 | Superior occipital gyrus | SOG | 135,136 | Mediodorsal medial magnocellular | tMDm |
| 55, 56 | Middle occipital gyrus | MOG | 137,138 | Mediodorsal lateral parvocellular | tMDl |
| 57, 58 | Inferior occipital gyrus | IOG | 139,140 | Lateral geniculate | tLGN |
| 59, 60 | Fusiform gyrus | FFG | 141,142 | Medial Geniculate | tMGN |
| 61, 62 | Postcentral gyrus | PoCG | 143,144 | Pulvinar anterior | tPuA |
| 63, 64 | Superior parietal gyrus | SPG | 145,146 | Pulvinar medial | tPuM |
| 65, 66 | Inferior parietal gyrus | IPG | 147,148 | Pulvinar lateral | tPuL |
| 67, 68 | Supramarginal gyrus | SMG | 149,150 | Pulvinar inferior | tPuI |
| 69, 70 | Angular gyrus | ANG | 151,152 | Anterior cingulate cortex, subgenual | ACCsub |
| 71, 72 | Precuneus | PCUN | 153,154 | Anterior cingulate cortex, pregenual | ACCpre |
| 73, 74 | Paracentral lobule | PCL | 155,156 | Anterior cingulate cortex, supracallosal | ACCsup |
| 75, 76 | Caudate nucleus | CAU | 157,158 | Nucleus accumbens | Nacc |
| 77, 78 | Lenticular nucleus, putamen | PUT | 159,160 | Ventral tegmental area | VTA |
| 79, 80 | Lenticular nucleus, pallidum | PAL | 161,162 | Substantia nigra, pars compacta | SNpc |
| 81, 82 | Thalamus | THA | 163,164 | Substantia nigra, pars reticulata | SNpr |
| 83, 84 | Heschl gyrus | HES | 165,166 | Red nucleus | RedN |
| 85, 86 | Superior temporal gyrus | STG | 167,168 | Locus coeruleus | LC |
| 87, 88 | Temporal pole: superior | TPOsup | 169 | Raphe nucleus, dorsal | RapheD |
| 89, 90 | Middle temporal gyrus | MTG | 170 | Raphe nucleus, median | RapheM |

**Table S2.** Detailed raw scores and correlated Z scores of separate neuropsychological assessments for both CN and SCD subjects.

| Characteristics | | CN | SCD | T-value(χ^2^) | p-value |
| --- | --- | --- | --- | --- | --- |
|  |  | n=74 | n=56 |  |  |
| Episodic memory | | | | | |
| AVLT-IR | raw score | 18.81(4.25) | 18.39(4.20) | 0.688 | 0.493 |
|  | Z score | 0.26(0.89) | 0.17(0.88) | 0.688 | 0.493 |
| AVLT-5MIN-DR | raw score | 6.08(2.13) | 6.14(2.21) | 0.192 | 0.848 |
|  | Z score | 0.22(0.88) | 0.25(0.91) | 0.192 | 0.848 |
| AVLT-20MIN-DR | raw score | 5.93(2.13) | 6.17(2.32) | -0.435 | 0.664 |
|  | Z score | 0.26(0.79) | 0.36(0.86) | -0.435 | 0.664 |
| LMT-IR | raw score | 6.27(3.01) | 5.62(3.01) | 0.988 | 0.325 |
|  | Z score | 0.32(0.99) | 0.11(0.99) | 0.988 | 0.325 |
| LMT-20MIN-DR | raw score | 4.91(2.80) | 4.67(2.73) | 0.268 | 0.789 |
|  | Z score | 0.27(1.01) | 0.19(0.99) | 0.268 | 0.789 |
| ROCFT-20MIN-DR | raw score | 16.28(6.15) | 17.26(6.18) | -1.289 | 0.200 |
|  | Z score | 0.14(0.90) | 0.28(0.91) | -1.289 | 0.200 |
| Visuospatial function | | | | | |
| ROCFT | raw score | 34.87(1.94) | 34.67(1.82) | 0.475 | 0.636 |
|  | Z score | 0.20(0.76) | 0.12(0.71) | 0.475 | 0.636 |
| CDT | raw score | 9.36(1.25) | 9.37(1.01) | -0.185 | 0.854 |
|  | Z score | 0.16(1.02) | 0.16(0.82) | -0.185 | 0.854 |
| Information Processing Speed | | | | | |
| DSST | raw score | 42.93(11.26) | 40.58(11.56) | 0.934 | 0.352 |
|  | Z score | 0.31(0.98) | 0.10(1.01) | 0.934 | 0.352 |
| TMT-A | raw score | 55.25(18.20) | 56.50(14.57) | 0.320 | 0.749 |
|  | Z score (r_TMT-A) | 0.24(1.09) | 0.08(0.82) | 0.427 | 0.670 |
| Stroop-A | raw score | 25.47(4.95) | 25.87(4.84) | -0.512 | 0.610 |
|  | Z score (r_Stroop-A) | 0.18(0.96) | 0.10(1.00) | 0.529 | 0.598 |
| Stroop-B | raw score | 42.12(10.19) | 41.94(10.49) | -0.032 | 0.974 |
|  | Z score (r_Stroop-B) | 0.14(0.94) | 0.16(0.92) | 0.013 | 0.990 |
| Executive function | | | | | |
| VFT | raw score | 21.72(13.81) | 20.46(4.79) | 0.432 | 0.667 |
|  | Z score | 0.21(1.42) | 0.08(0.49) | 0.432 | 0.667 |
| DST | raw score | 12.68(1.99) | 12.82(1.83) | -0.777 | 0.438 |
|  | Z score | 0.16(0.98) | 0.23(0.90) | -0.777 | 0.438 |
| TMT-B | raw score | 126.43(32.30) | 139.39(39.51) | -1.343 | 0.182 |
|  | Z score(r_TMT-B) | 0.33(0.87) | 0.08(0.96) | 0.910 | 0.364 |
| Stroop-C | raw score | 79.43(22.15) | 78.55(23.98) | 0.257 | 0.798 |
|  | Z score(r_Stroop-C) | 0.10(0.92) | 0.18(0.99) | -0.578 | 0.564 |
| Similarity | raw score | 19.24(3.69) | 19.37(3.28) | -0.708 | 0.480 |
|  | Z score | 0.31(0.82) | 0.34(0.73) | -0.708 | 0.480 |

Data are presented as mean (standard deviation, SD). To note, reversed *Z* scores of TMT-A/B and Stroop-A/B/C were applied (*i.e.*, r_TMT-A/B; r_Stroop-A/B/C) to further calculate the composite Z scores of cognitive domains. We applied our in-home MATLAB code to regress out covariates (*i.e.,* age, gender and educational level). No significant difference was found between CN and SCD subjects. Abbreviations: AVLT-IR, Auditory Verbal Learning Test-immediate recall; AVLT-5-min-DR, Auditory Verbal Learning Test-5-minute delayed recall; AVLT-20-min-DR, Auditory Verbal Learning Test-20-minute delayed recall; LMT-IR, Logical Memory Test-immediate recall; LMT-20-min-DR, Logical Memory Test-20-minute delayed recall; ROCFT-20min-DR, Rey-Osterrieth Complex Figure Test-20-minute delayed recall; CDT, Clock Drawing Test; ROCFT, Rey-Osterrieth Complex Figure Test; DSST, Digital Symbol Substitution Test; TMT-A, Trail Making Test-A; Stroop, Stroop Color and Word Test; VFT, Verbal Fluency Test; DST, Digit Span Test; TMT-B, Trail Making Test-B; Similarity, Semantic Similarity Test; CN, normal controls; SCD, subjective cognitive decline.

**Table S3. Regions of Interest (ROI) in brain-wide voxel-based analyses (BWAS).**

| **No.** | **L/R** | **Regions** | **Voxels in ROI** | **Peak MA value** | **MNI (Peak)** |
| --- | --- | --- | --- | --- | --- |
| **CN *v.s.* SCD** | | | | | |
| ROI1 | R | Precentral_R | 24 | 752 | 46 -18 68 |
| ROI2 | R | Rolandic_Oper_R | 14 | 148 | 50 -6 12 |
| ROI3 | R | Insula_R | 18 | 107 | 46 6 -4 |
| ROI4 | R | Calcarine_R | 12 | 289 | 10 -94 12 |
| ROI5 | R | Cuneus_R | 25 | 541 | 6 -94 16 |
| ROI6 | R | Occipital_Sup_R | 21 | 360 | 14 -98 16 |
| ROI7 | L | Occipital_Mid_L | 40 | 190 | -34 -66 28 |
| ROI8 | R | Occipital_Mid_R | 28 | 223 | 30 -94 16 |
| ROI9 | R | Postcentral_R | 20 | 116 | 50 -22 48 |
| ROI10 | L | Parietal_Sup_L | 66 | 282 | -14 -62 44 |
| ROI11 | L | SupraMarginal_L | 19 | 66 | -62 -34 24 |
| ROI12 | L | Angular_L | 20 | 105 | -38 -54 24 |
| ROI13 | L | Precuneus_L | 23 | 304 | -10 -62 52 |
| ROI14 | R | Heschl_R | 10 | 112 | 50 -10 8 |
| ROI15 | L | Temporal_Mid_L | 55 | 249 | -46 -58 4 |

Regions with significant voxel numbers ≥10 without any correction were presented. Positive measure of association (MA) indicates strengthened functional connectivity links in CNs compared with SCD patients. Abbreviations: CN, normal control; SCD, subjective cognitive decline.

**SI Figures**


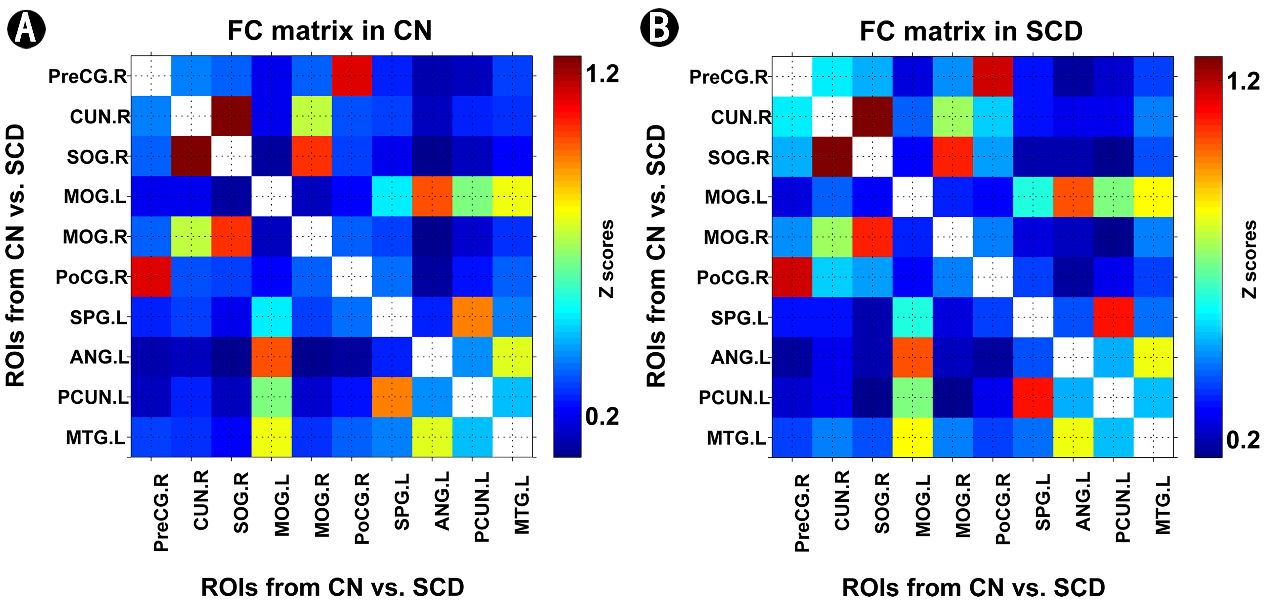


**Fig S1. FC matrixes for CN and SCD participants through ROI-wise FC analysis.**

Color bars represent the Z scores of altered FC among matched ROIs. Abbreviations: FC, functional connectivity; CN, normal controls; SCD, subjective cognitive decline; PreCG, precentral gyrus; CUN, cuneus; SOG, superior occipital gyrus; MOG, middle occipital gyrus; PoCG, postcentral gyrus; SPG, superior parietal gyrus; ANG, angular gyrus; PCUN, precuneus; MTG, middle temporal gyrus; L, left; R, right.


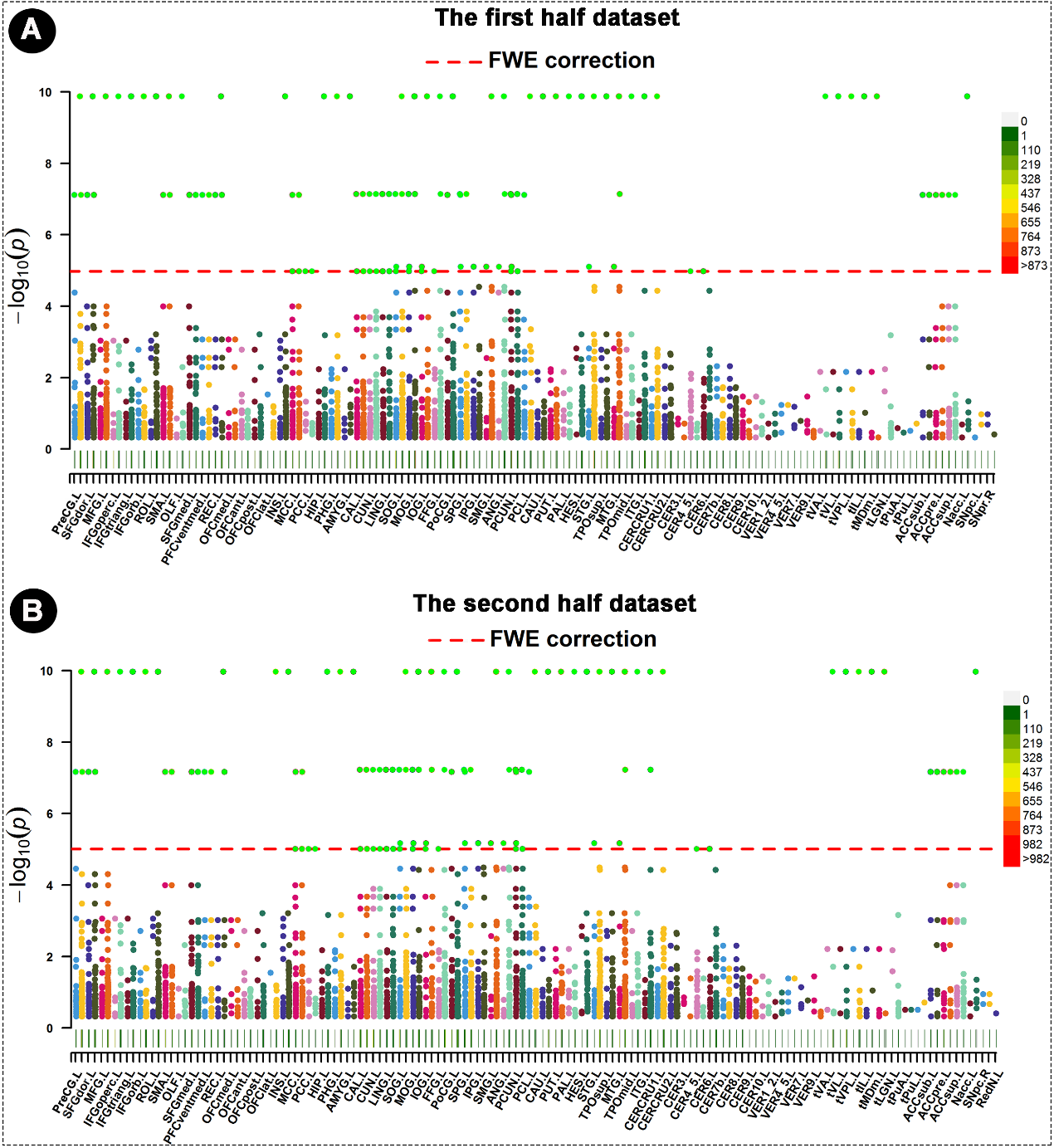


**Fig S2.** Manhattan plot presenting the probability values of altered FC links in SCD patients for both datasets (FWE correction, 0.05, MA >40, cluster size >30 voxels for the first half and the second half dataset). Every dot represents a FC link of two matched voxels. The red line indicates FWE correction with a p value < 3.33×10-2. The AAL3 atlas was applied to describe the specific location of voxels, with its details listed in SI Tables S1.


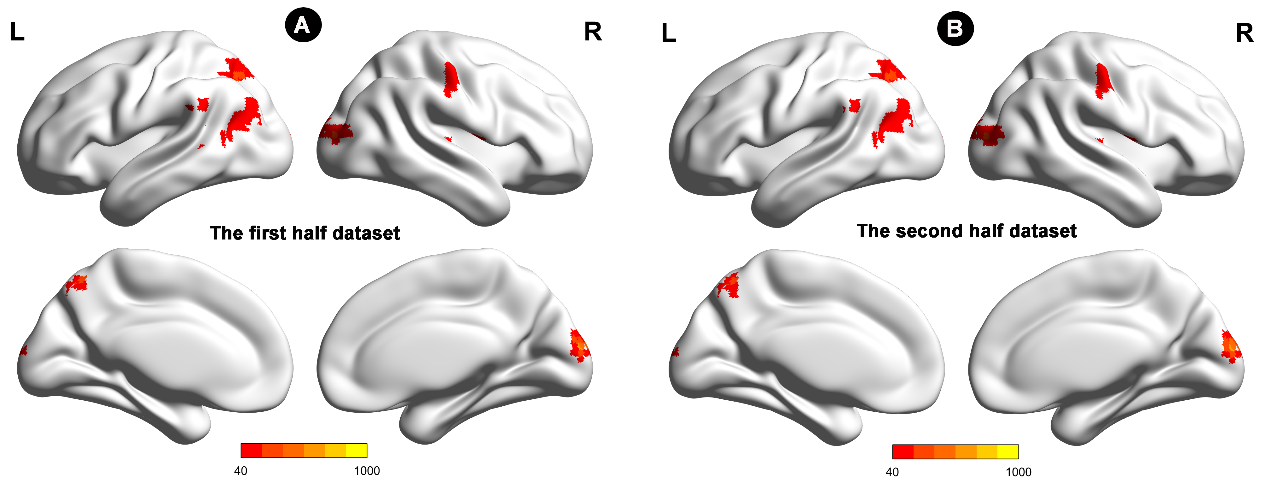


**Fig S3.** The locations of all voxels in the brain that have significantly different functional connectivities between the SCD and the CN for both datasets (FWE correction, 0.05, MA >40, cluster size >30 voxels for the first half and the second half dataset).

**References**

Ashburner, J., and Friston, K.J. (2009). Computing average shaped tissue probability templates. *Neuroimage* 45**,** 333-341.

Brady, R.O., Jr., Gonsalvez, I., Lee, I., Ongur, D., Seidman, L.J., Schmahmann, J.D., Eack, S.M., Keshavan, M.S., Pascual-Leone, A., and Halko, M.A. (2019). Cerebellar-Prefrontal Network Connectivity and Negative Symptoms in Schizophrenia. *Am J Psychiatry***,** appiajp201818040429.

Cedres, N., Machado, A., Molina, Y., Diaz-Galvan, P., Hernandez-Cabrera, J.A., Barroso, J., Westman, E., and Ferreira, D. (2019). Subjective Cognitive Decline Below and Above the Age of 60: A Multivariate Study on Neuroimaging, Cognitive, Clinical, and Demographic Measures. *J Alzheimers Dis* 68**,** 295-309.

Chen, J., Chen, G., Shu, H., Chen, G., Ward, B.D., Wang, Z., Liu, D., Antuono, P.G., Li, S.J., Zhang, Z., and Alzheimer's Disease Neuroimaging, I. (2019a). Predicting progression from mild cognitive impairment to Alzheimer's disease on an individual subject basis by applying the CARE index across different independent cohorts. *Aging (Albany NY)* 11**,** 2185-2201.

Chen, J., Chen, R., Xue, C., Qi, W., Hu, G., Xu, W., Chen, S., Rao, J., Zhang, F., and Zhang, X. (2022). Hippocampal-Subregion Mechanisms of Repetitive Transcranial Magnetic Stimulation Causally Associated with Amelioration of Episodic Memory in Amnestic Mild Cognitive Impairment. *J Alzheimers Dis* 85**,** 1329-1342.

Chen, J., Duan, X., Shu, H., Wang, Z., Long, Z., Liu, D., Liao, W., Shi, Y., Chen, H., and Zhang, Z. (2016a). Differential contributions of subregions of medial temporal lobe to memory system in amnestic mild cognitive impairment: insights from fMRI study. *Sci Rep* 6**,** 26148.

Chen, J., Ma, N., Hu, G., Nousayhah, A., Xue, C., Qi, W., Xu, W., Chen, S., Rao, J., Liu, W., Zhang, F., and Zhang, X. (2020). rTMS modulates precuneus-hippocampal subregion circuit in patients with subjective cognitive decline. *Aging (Albany NY)* 12.

Chen, J., Shu, H., Wang, Z., Liu, D., Shi, Y., Zhang, X., and Zhang, Z. (2015). The interaction of APOE genotype by age in amnestic mild cognitive impairment: a voxel-based morphometric study. *J Alzheimers Dis* 43**,** 657-668.

Chen, J., Shu, H., Wang, Z., Zhan, Y., Liu, D., Liao, W., Xu, L., Liu, Y., and Zhang, Z. (2016b). Convergent and divergent intranetwork and internetwork connectivity patterns in patients with remitted late-life depression and amnestic mild cognitive impairment. *Cortex* 83**,** 194-211.

Chen, J., Shu, H., Wang, Z., Zhan, Y., Liu, D., Liu, Y., and Zhang, Z. (2019b). Intrinsic connectivity identifies the sensory-motor network as a main cross-network between remitted late-life depression- and amnestic mild cognitive impairment-targeted networks. *Brain Imaging Behav*.

Chen, J., Yan, Y., Gu, L., Gao, L., and Zhang, Z. (2019c). Electrophysiological Processes on Motor Imagery Mediate the Association Between Increased Gray Matter Volume and Cognition in Amnestic Mild Cognitive Impairment. *Brain Topogr*.

Dunn, C.J., Duffy, S.L., Hickie, I.B., Lagopoulos, J., Lewis, S.J., Naismith, S.L., and Shine, J.M. (2014). Deficits in episodic memory retrieval reveal impaired default mode network connectivity in amnestic mild cognitive impairment. *Neuroimage Clin* 4**,** 473-480.

Friston, K.J., Williams, S., Howard, R., Frackowiak, R.S., and Turner, R. (1996). Movement-related effects in fMRI time-series. *Magn Reson Med* 35**,** 346-355.

Hao, L., Wang, X., Zhang, L., Xing, Y., Guo, Q., Hu, X., Mu, B., Chen, Y., Chen, G., Cao, J., Zhi, X., Liu, J., Li, X., Yang, L., Li, J., Du, W., Sun, Y., Wang, T., Liu, Z., Liu, Z., Zhao, X., Li, H., Yu, Y., Wang, X., Jia, J., and Han, Y. (2017). Prevalence, Risk Factors, and Complaints Screening Tool Exploration of Subjective Cognitive Decline in a Large Cohort of the Chinese Population. *J Alzheimers Dis* 60**,** 371-388.

Jessen, F., Amariglio, R.E., Van Boxtel, M., Breteler, M., Ceccaldi, M., Chetelat, G., Dubois, B., Dufouil, C., Ellis, K.A., Van Der Flier, W.M., Glodzik, L., Van Harten, A.C., De Leon, M.J., Mchugh, P., Mielke, M.M., Molinuevo, J.L., Mosconi, L., Osorio, R.S., Perrotin, A., Petersen, R.C., Rabin, L.A., Rami, L., Reisberg, B., Rentz, D.M., Sachdev, P.S., De La Sayette, V., Saykin, A.J., Scheltens, P., Shulman, M.B., Slavin, M.J., Sperling, R.A., Stewart, R., Uspenskaya, O., Vellas, B., Visser, P.J., Wagner, M., and Subjective Cognitive Decline Initiative Working, G. (2014). A conceptual framework for research on subjective cognitive decline in preclinical Alzheimer's disease. *Alzheimers Dement* 10**,** 844-852.

Petersen, R.C., Smith, G.E., Waring, S.C., Ivnik, R.J., Tangalos, E.G., and Kokmen, E. (1999). Mild cognitive impairment: clinical characterization and outcome. *Arch Neurol* 56**,** 303-308.

Power, J.D., Barnes, K.A., Snyder, A.Z., Schlaggar, B.L., and Petersen, S.E. (2012). Spurious but systematic correlations in functional connectivity MRI networks arise from subject motion. *Neuroimage* 59**,** 2142-2154.

Rolls, E.T., Huang, C.C., Lin, C.P., Feng, J., and Joliot, M. (2020). Automated anatomical labelling atlas 3. *Neuroimage* 206**,** 116189.

Van Dijk, K.R., Sabuncu, M.R., and Buckner, R.L. (2012). The influence of head motion on intrinsic functional connectivity MRI. *Neuroimage* 59**,** 431-438.

Winblad, B., Palmer, K., Kivipelto, M., Jelic, V., Fratiglioni, L., Wahlund, L.O., Nordberg, A., Backman, L., Albert, M., Almkvist, O., Arai, H., Basun, H., Blennow, K., De Leon, M., Decarli, C., Erkinjuntti, T., Giacobini, E., Graff, C., Hardy, J., Jack, C., Jorm, A., Ritchie, K., Van Duijn, C., Visser, P., and Petersen, R.C. (2004). Mild cognitive impairment--beyond controversies, towards a consensus: report of the International Working Group on Mild Cognitive Impairment. *J Intern Med* 256**,** 240-246.

Xue, C., Yuan, B., Yue, Y., Xu, J., Wang, S., Wu, M., Ji, N., Zhou, X., Zhao, Y., Rao, J., Yang, W., Xiao, C., and Chen, J. (2019). Distinct Disruptive Patterns of Default Mode Subnetwork Connectivity Across the Spectrum of Preclinical Alzheimer's Disease. *Front Aging Neurosci* 11**,** 307.

Yan, C.G., Craddock, R.C., Zuo, X.N., Zang, Y.F., and Milham, M.P. (2013). Standardizing the intrinsic brain: towards robust measurement of inter-individual variation in 1000 functional connectomes. *Neuroimage* 80**,** 246-262.

Yan, C.G., Wang, X.D., Zuo, X.N., and Zang, Y.F. (2016). DPABI: Data Processing & Analysis for (Resting-State) Brain Imaging. *Neuroinformatics* 14**,** 339-351.

Yan, T., Wang, W., Yang, L., Chen, K., Chen, R., and Han, Y. (2018). Rich club disturbances of the human connectome from subjective cognitive decline to Alzheimer's disease. *Theranostics* 8**,** 3237-3255.
